# Comparative Analysis of Saliva and Plasma Proteins Patterns in Pregnant Cows—Preliminary Studies

**DOI:** 10.3390/ani12202850

**Published:** 2022-10-20

**Authors:** Wioleta Mojsym, Jacek Wawrzykowski, Monika Jamioł, Łukasz Chrobak, Marta Kankofer

**Affiliations:** Department of Biochemistry, Faculty of Veterinary Medicine, University of Life Science in Lublin, Akademicka 12, 20-033 Lublin, Poland

**Keywords:** proteomics, mass spectrometry, apolipoproteins, cattle, saliva, pregnancy

## Abstract

**Simple Summary:**

One of the most crucial topics about cattle breeding is pregnancy. During this state, there are many changes in protein expression and abundance. These changes find reflection not only in plasma protein patterns but also in saliva, which is easier to obtain than blood. The aim of this study was the analysis of plasma and salivary protein profiles in pregnant cows in order to search for valuable markers of pregnancy status. In this study, the presence of apolipoproteins possibly related to bovine pregnancy was confirmed both in plasma and saliva. This means that saliva can be considered a good source of information about the condition of the organism, including during pregnancy. It is possible that the comparison of salivary and plasma proteomes can be a helpful tool to assess the pregnancy status of cattle, and can be useful for developing rapid tests from saliva.

**Abstract:**

Pregnancy is a physiological state that can be described, from a biochemical point of view, using protein patterns. The present study focused on the comparison of protein patterns between the saliva and plasma of pregnant cows to search for possible markers which are present both in plasma and saliva. Saliva and plasma were collected from healthy, pregnant (3–4 months) and non-pregnant (C; *n* = 4) cows aged between 4 and 8 years (P; *n* = 8) from the same farm. Biological material was analyzed using 2D electrophoresis and MS identification. Among identified spots, there were those which could be related to pregnancy (e.g., apolipoproteins I and II in all examined matrices or transforming growth factor-beta-induced protein ig-h3 in albumin-free plasma) as well as those which are responsible for regulating of cellular processes (e.g., pyruvate kinase and aspartate aminotransferase in all examined matrices, or lactate dehydrogenase, phosphoglycerate kinase, and NADH dehydrogenase in plasma). Further identification of common spots and those only specific to saliva as well as the comparison between other periods of pregnancy are necessary; it is already clear that saliva can be considered a valuable diagnostic matrix containing potential markers of physiological and pathological status.

## 1. Introduction

One of the areas of interest for researchers in the field of cattle breeding is pregnancy. Pregnancy in dairy cows is not only a source of calves, which are used for the development of breeding and repair of the herd, but also maintains lactation in order to obtain milk for the dairy industry. For this reason, it is important from both a breeding and veterinary point of view to deepen our knowledge not only of the development of pregnancy but also of changes in physiological parameters in cows. This may result in the selection of specific biomarkers that will be an alternative to conventional methods of pregnancy recognition in cattle or confirmation of the outcome of these methods, as well as for the assessment of possible pathologies of pregnancy [1]. There are also other applications for saliva in diagnostics; for example, biomarkers from saliva may begin to be examined for oral squamous cell carcinoma diagnosis [2]. In buffalo, there are attempts to detect estrous time using biomarkers from saliva for planning insemination in more precise terms [3]. Studies that examined the usefulness of saliva biomarkers in the diagnosis of equine gastric ulcer syndrome (EGUS), equine glandular gastric disease (EGGD), and equine squamous gastric disease (ESGD) were also performed [4]. Moreover, new, increasingly sensitive, and more specific methods for plasma, saliva, or other fluids biomarkers detection are being developed [5].

For many years, the efforts of researchers have focused mainly on characterizing the composition of blood plasma in both humans and animals, and changes in the composition of these biological fluids depending on the physiological or pathological state of the organism. These efforts have contributed significantly to the development of laboratory diagnostics for both human and veterinary medicine, and many parameters routinely determined in plasma allow the location of the disease process in the body, as well as the confirmation of the diagnosis or evaluation of the treatment efficiency [2,4,6,7,8].

However, in recent years, the development of laboratory techniques has occurred, including proteomic techniques, enabling global analysis of the proteome in a given biological material and allowing for the accurate identification of proteins including potential biomarkers [6,9,10] in various physiological or pathological states. This development increased the interests of the scientific community in the search for new, more easily available diagnostic fluids that are sources of biomarkers (including proteome-based biomarkers) to monitor general health, animal welfare, and early disease detection as well as to assess the safety and quality of animal products [11]. In the case of livestock animals, especially cattle, as biological fluids with potential diagnostic use and the source of biomarkers, milk [6,12,13] and saliva [14,15] are mainly considered.

Due to the fact that saliva is a combination of both local (from the salivary glands) and systematic components (derived from blood) [15], it provides a potential source of information about the systemic processes occurring in the body which will allow for the evaluation of the physiological or pathological state of the organism. However, the use of saliva in the diagnosis of animal diseases first requires a thorough analysis of its protein composition under different conditions [15] because the protein composition of saliva—compared to plasma—is heterogeneous and shows inter- and intra-individual variability depending on factors such as age, feed, environmental conditions, time of day, or the physiological condition of the animal [16,17,18].

Currently, bovine pregnancy is diagnosed by rectal palpation, ultrasonography, or by the determination of changes in the concentration of progesterone in blood. Moreover, rapid molecular tests based on the presence of PAGs and DG-29 in the blood are also recently available to detect pregnancy. Barbato et al. [19,20], as well as De Carolis et al. [21], described the changes in PAG in the plasma of cows during pregnancy and the postpartum period. Unfortunately, due to the wide range of changes in the plasma PAG concentration, these tests may not detect the early stage of pregnancy (when the increase in concentration is below the value that persists after childbirth in the plasma). Thus, efforts are still being made to identify potential plasma biomarkers that confirm pregnancy in a less invasive and less stressful manner [13]. Nevertheless, similar efforts are not made regarding saliva as a potential diagnostic material not only for animal diseases or pathological conditions but also for the diagnosis of physiological conditions such as pregnancy.

The aim of our study was to characterize and attempt to compare the proteomic profile of saliva and plasma of pregnant dairy cows in order to search for markers visible in both plasma and saliva. The value of these markers was established by the comparison with control–non-pregnant cows. The results of our study will contribute to the increase of interest in the search for pregnancy-specific proteins in the saliva of animals because saliva as a research material is easy to obtain in a minimally invasive way, even in many repetitions, without stressing the animals.

## 2. Materials and Methods

### 2.1. Animals

Both saliva and plasma were collected during routine veterinary examinations using good veterinary practice from healthy, pregnant Holstein-Friesian cows (P; *n* = 8). The progress of pregnancy was evaluated by the date of artificial insemination and per rectal USG and established for 3–4 months. The control (non-pregnant) group was obtained from the same farm (C; *n* = 4). Animals were at a similar age as pregnant individuals (4–8 years old). Selected cows were at least 60 days after the last parturition [19,20,21]. Efforts were made in order to obtain the most homogenous group with regard to feeding and milk production. All samples were collected in May, on the same day in the morning.

### 2.2. Saliva and Blood Collection and Processing

Saliva samples were collected once per animal on the same day in the morning before feeding to avoid any contamination of saliva with food. Saliva was collected using sponges which were mounted in the space between teeth and cheeks. The sponges were placed in test tubes (15 mL), and centrifuged at 1000× *g*, at 20 °C for 10 min (MPW-150 R; Warsaw, Poland) to obtain biological material. The obtained material was portioned (200 µL) and frozen at −20 °C until analysis.

Blood was collected immediately after saliva collection from the jugular vein into tubes coated with EDTA as an anticoagulant (Vacutest, Italy). Samples were stored at 4 °C in transport and centrifuged within 2 h after collection (1000× *g* at 20 °C for 15 min; MPW-150 R; Warsaw, Poland). Immediately after centrifugation, aliquots were portioned (200 µL) and frozen at −20 °C until the next steps.

The samples were analyzed in duplicate.

### 2.3. Two-Dimensional Analysis

The removal of albumin from plasma samples.

The procedure for the removal of albumin from plasma samples from cows was based on the Aurum Affi-Gel Blue columns, Bio-Rad. In summary, columns were washed twice with PBS and then loaded with 400 µL of 4× diluted plasma. Unbound proteins were washed from the columns with 400 µL PBS; the bound albumin was eluted with 400 µL rehydration solution (7 M urea, 2 M thiourea, 1% ASB-14, 40 mM TRIS, 0.001% bromophenol blue). Measurement of all protein concentrations in the samples was performed using the Bradford method [22] (Bradford reagent, Sigma, Poznań, Poland). The plasma samples and plasma without albumin were analyzed individually and saliva samples, due to the low protein content, were pooled in pairs and concentrated on a VivaSpin column (cut-off 3 kDa, Sartorius, Göttingen, Germany).

### 2.4. Isoelectric Focusing

Immediately before, the isoelectric focusing sample was diluted to 200 µL in the rehydration buffer (up to a concentration of 0.35 mg/mL protein). The samples were loaded onto IPG strips (4–7 pH, 11 cm length) and the process was carried out in the Protean IEF cell (active rehydration at 50 V, 12 h, focusing conditions: 8000 V up to 35,000 V-h, a maximum current of 50 µA/IPG strip) (Bio-Rad, Warszawa, Poland).

Before loading onto SDS-polyacrylamide gels, IPG strips were incubated for 15 min in an equilibration buffer (50 mM Tris-HCl, pH 8.8, 6 M urea, 30% glycerol, 2% SDS) containing 5 mM DTT and then for another 15 min in equilibration buffer containing 15 mM iodoacetamide. The SDS-polyacrylamide gels (20 × 20 cm, 1.5 mm, T = 11%, C = 2.6%) were proceeded in accordance with Laemmli (1970) [23]. The second dimension was performed using a Protean II according to the manufacturer’s instructions (Bio-Rad, Warsaw, Poland).

### 2.5. Gels Staining and Analysis

For manual removal of spots of interest, protein silver nitrate staining of 2D gels was performed according to mass spectrometry compatible protocol [24]. The stained gels were scanned on LabScan software using a densitometer calibration curve (Imagescanner III, GE Healthcare, Warszawa, Poland). The gel image analysis was then performed in Delta2D software (Decodon, Greifswald, Germany). Briefly, the optical density of the spots on the gels from the trials was compared and a statistical comparison was made between the examined groups. In order to ensure reliable results, a fused master gel was made from the pool of all gels used for the analysis (*n* = 36). The individual gels were statistically analyzed using nonparametric statistical tests: Wilcoxon and Kruskal–Wallis, a probability *p* < 0.05 was taken to be statistically significant.

### 2.6. Mass Spectrometry-Based Protein Identification

The spots of interest were excised from the gels, chopped into pieces, and transferred into 0.5 mL tubes. In-gel digestion of proteins for MS was prepared according to the following protocol: the enzymatic digestion of the proteins was made at 37 °C overnight with 10 μL of 12.5 ng/mL trypsin (Trypsin Gold, Mass Spectrometry Grade, Promega, Medison, WI, USA). After digestion, the peptides were extracted 3 times with 50 μL 70% acetonitrile with 1.5% TFA by sonification for 15 min at room temperature in an ultrasonic water bath (UltronU-507, Ultron, Dywity, Poland). The supernatant was collected and dried in the Concentrator plus (Eppendorf) for 45 min at 40 °C.

The peptide pellet was allowed to reswell in 10 μL of 0.1% TFA and purified with μC18 ZipTip (Eppendorf, Poznań, Poland) according to the manufacturer’s instructions. The cleaned peptide mixture (1 μL) was pipetted to prespotted HCCA-PAC (with 3,5-dimethoxy-4-hydroxycinnamic acid) target frame (Bruker, Poznań, Poland) and allowed to dry at room temperature. Mass spectra were acquired with an Ultraflex III MALDI TOF/TOF spectrometer (Bruker, Poznań, Poland). The acquisition was performed in positive ion reflector mode with a 25-kV acceleration voltage. External calibrations were performed using the peptide calibration standard (Peptide Calibration Standard II, Bruker, Poznań, Poland). Flex analysis 3.0 software Bruker Daltonics was used for the selection of the monoisotopic peptide masses.

The obtained mass spectra were analyzed using the Flex Analysis 3.0 software (Bruker, Bremen, Germany). The list of peaks was generated in the range of 700–4000 *m*/*z* for the signal-to-noise ratio greater than 10. The resulting list of peaks was used to identify the protein by comparison with the entries of the database using the Mascot 2.2 software (Matrix Science Ltd., London, UK) with the database Swiss-Prot (SwissProt release 2022_02) restricted to the “other Mammalia” taxonomy. Search parameters were set as follows: enzyme–trypsin, modification obligatory–carboamoinodmethylation cysteine, possible modification–oxidation of methionine, error of 50 ppm. Mascot score has been calculated according to Perkins et al. [25] and Koenig et al. [26]; scores greater than 67 were considered significant (*p* < 0.05).

## 3. Results

**Figure 1:**
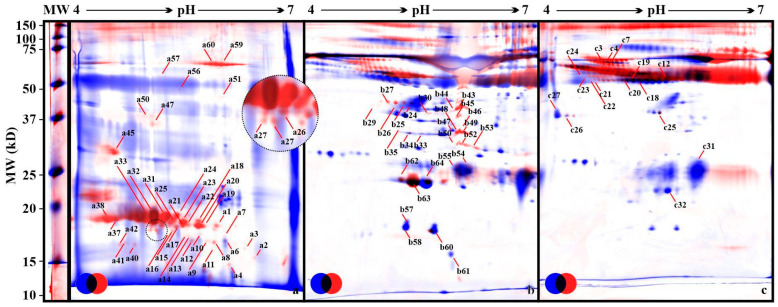
Fused gel images of the samples. Legend: (**a**) Saliva. (**b**) Plasma. (**c**) Albumin-free plasma, MW–broad range protein ladder. Arrows indicate identified proteins (see Tables S1–S3). Pool images from all samples in experiment *n* = 36. Control (C) group, blue; examined (P) group, red; common, black.

All gel images were processed by the Delta2D software (version 4.7.3 Final 05, Decodon, Greifswald, Germany). The results of the 2D separation of proteins from saliva and plasma samples are presented in Figure 1.

Out of 210 detected proteins in saliva, 119 (57%) differed significantly between saliva samples from pregnant and non-pregnant cows. In the plasma samples, there were 543 proteins. The comparison of proteins derived from plasma samples from pregnant and non-pregnant cows showed 324 (60%) significantly different spots. Out of 427 proteins found in albumin-free plasma samples, 136 (33%) were significantly different between pregnant and non-pregnant animals. The selection of spots for further identification was done based on the intensity of staining corresponding to the amount of protein in the examined samples. A total of 89 proteins were identified: 41 in saliva (Supplementary Table S1), 31 in plasma (Supplementary Table S2), and 17 spots in albumin-free plasma (Supplementary Table S3). The classification of proteins was made based on the available literature [27,28,29].

Identified saliva proteins represented various protein classes, including proteins classified as “plasma proteins” or “predicted secreted proteins”, but also proteins characterized as “predicted membrane proteins” and “predicted intracellular proteins” (source: https://www.proteinatlas.org/ (accessed on 8 October 2022), (Protein Atlas version 21.0, 18 November 2021). Of the 37 salivary proteins identified, 13 of them were classified as “plasma proteins”. Moreover, saliva proteins are largely composed of phosphoproteins and glycoproteins (source: https://david.ncifcrf.gov/ (accessed on 8 October 2022), (release DAVID 2021, December 2021).

The analysis of plasma, both albumin-purified and albumin-containing, revealed that these matrices also contain proteins that are both extra- and intracellular components (source: https://www.uniprot.org/ (accessed on 8 October 2022), (UniProt release 2011_09)). In the case of 7 proteins, they were confirmed in both saliva and plasma of pregnant cows: hemoglobin subunit beta, apolipoprotein A-II, methylmalonyl-CoA mutase mitochondrial, T-complex protein 1 subunit theta, putative helicase MOV-10, and apolipoprotein AI, as well as serum albumin (Supplementary Tables S1–S3). In addition, aspartate aminotransferase, which is a diagnostic plasma indicator enzyme, was also present in saliva.

## 4. Discussion

The present study focused on the comparison between plasma and saliva, collected from pregnant and non-pregnant cows at the same time, to search for possible markers of pregnancy status and the usefulness of saliva for diagnostic purposes. Identified proteins expressed only partial similarities in examined fluids.

Whole saliva is a complex biological fluid secreted to the initial part of the digestive tract—oral cavity—containing both components secreted by the salivary glands, as well as from the upper respiratory tract, oropharynx, gingival fluid, cell debris, esophagus, rumen, food debris, microorganisms, or those of blood origin [30,31]. It mainly consists of water (approximately 99.5%) but also contains several soluble organic components (including proteins, hormones, and metabolites) as well as inorganic electrolytes [14,18].

The complete composition of saliva has been known for a long time and was described elsewhere [14,17], but only the current progress in the field of proteomics has made it possible to characterize the protein profile of cow saliva [14] and small ruminants [32], as well as pigs [33], or also human [34,35,36,37,38,39]. However, it should be kept in mind that saliva expresses a “dynamic proteome” showing high protein pattern instability under the influence of many factors. For that reason, it is necessary to assess changes in protein composition in relation to various physiological and pathological conditions, which is the first necessary step that will allow the future selection of potential biomarkers of health status or disease.

In the case of bovine whole saliva, 402 proteins and 45 N-linked glycoproteins have been identified. Moreover, according to others [15,30], it is worth noting that saliva is an easily available, non-invasive, and stressless research material that can be collected repeatedly without violating animal welfare.

Research carried out by Yan and colleagues (2009) [40] led to the identification of 1444 proteins found in human whole saliva, which shows the “richness” of proteins in this inconspicuous biological fluid and the powerful diagnostic potential.

Our study focused on a comparison of the salivary and plasma proteins of the untreated and non-albumin fractions of plasma originating from cows. We focused on a pH range between 4 and 7 which gave the possibility to separate selected molecules. According to our best knowledge, these efforts are the first attempt to compare the protein composition of these biological fluids in cattle, especially in relation to pregnancy. In the case of human tests, such a comparison was carried out in healthy adults [40,41].

The period of 3–4 months of pregnancy was selected in this study due to its importance for pregnancy course. At this time, we are dealing with the developing fetus as well as with the developed placenta because during the period of about 90–255 days of pregnancy the number of cotyledons is constant while their surface increases [42]. Moreover, in the first trimester of pregnancy, there is a development of a complex loop of terminal capillaries within the stroma of the uterine caruncle in the vicinity of the crypts [43] which allows the substance exchange between the fetus and the body of the mother and may be a source of biomarkers in the blood testifying to the “presence of the fetus”. Moreover, due to the possibility of passing the substance from the bloodstream through a thin layer of epithelial cells that separates the blood from the salivary ducts [30], these molecules can appear in saliva.

The analysis of identified proteins in examined matrices showed that saliva only partly reflects the content of plasma in our conditions of the experiment (time of sample collection, conditions of protein separation). The functions of the remaining identified proteins are mentioned in respective tables. Our results confirmed the presence of apolipoproteins in saliva which can be related to the pregnancy course and some parameters involved in regular metabolism. For example, vacuolar protein-sorting-associated protein 36, present in plasma samples collected from pregnant individuals, was not present in non-pregnant cows. That difference is possible in connection with increased cell proliferation during pregnancy, which has been tested in mice [44]. In pregnant cattle, as opposed to non-pregnant, hemoglobin subunit beta was found as well as a different expression of apolipoprotein A-I—overlapping with observations made by Szczubiał M. et al. (2017) in plasma coming from pregnant and non-pregnant dogs [45]. Tuftelin-interacting protein 11, probably a splicing factor, also appears only in the plasma of pregnant cattle [46]. A similar situation is with general transcription factor IIF subunit 2 [47] and translation initiation factor eIF-2B subunit beta [48]. Annexin A9 linked to early embryonic development was found only in samples derived from pregnant individuals. The same protein was discovered earlier in material collected from the pig uterus from a peri-implantation period [49].

Other researchers [50] compared salivary protein profiles during different stages of the estrous cycle in buffalo. Proteins were fractionated by bRP-HPLC and identified in a captive spray-Maxis-HD q-TOF mass spectrometer. The authors of the present experiment obtained a more complete protein profile than in the study of Shashikumar et al. [50] due to the use of electrophoretic techniques combined with MALDI-TOF. This combination has been used in earlier studies and is a powerful tool to characterize the salivary and plasma proteome [15,18].

Our last studies on the antioxidative status of saliva in cows of different ages similarly showed only a partial reflection of the content of plasma with regard to antioxidative/oxidative parameters; but it showed age-related differences which can be used in the description of the physiological status of cows [51].

Human studies, however, provided many examples of the use of saliva parameters as diagnostic tools in the estimation of delivery time [52,53].

Due to the fact that plasma has been the subject of studies for researchers for many years, the proteomic plasma pattern is well-described in the literature for cattle [10,54], as well as other animals [7,8] and humans, and the use of blood parameters determinations in medical practice is common in both human and veterinary medicine. In addition, for cattle, efforts are made to determine the changes in the plasma protein profile during late pregnancy [28,55] and attempts are made to identify potential pregnancy-specific biomarkers in the plasma [56,57]. Similar efforts are also made in the case of dogs [45].

Our previous studies on the plasma proteins around parturition in cows showed that, among identified proteins, there were 15 spots that differed significantly between patterns detected at parturition and −14 days; but metalloproteinase inhibitor, collagen alpha II chain, and LDH seemed to be the most promising molecules considered as parturition markers due to their functions [29]. The present study confirmed the presence of LDH in the plasma of early pregnant cows.

Interestingly, some researchers are looking for pregnancy-specific proteins in the milk of cows [13] or even in urine [58] enabling early diagnosis of pregnancy as an alternative to conventional stress and invasive methods currently used in veterinary practice, confirmation of their outcome, or monitoring of pregnancy.

## 5. Conclusions

Due to the fact that the ultimate goal of identifying and characterizing the biological fluids is to use this information in the future for, among others, health screening and the detection of diseases, we hope that the results of our research will attract the attention of the scientific community dealing with livestock animals science to the enormous potential of saliva as an easy and a good source of information about the state of the body. In our opinion, comparing salivary proteome with plasma proteome, which has often revealed their value in clinical application, will help identify salivary-specific biomarkers, as well as plasma-derived biomarkers present in saliva in assessing both pregnancy and the health status and welfare of the cattle.

## Data Availability

Raw data are available by the first author upon request.

## Supplementary Materials

The following supporting information can be downloaded at: https://www.mdpi.com/article/10.3390/ani12202850/s1, Table S1: Identification of saliva proteins; Table S2: Identification of plasma proteins; Table S3: Identification of proteins from albumin-free fraction.
