# Supplementary material for: Comparative Analysis of Saliva and Plasma Proteins Patterns in Pregnant Cows—Preliminary Studies
Source: Animals (Basel). 2022 Oct 20;12(20):2850. doi: 10.3390/ani12202850 (PMC9597767; doi:10.3390/ani12202850)
Supplement: Supplementary file 1 [file animals-12-02850-s001.zip › Table S1 Identification of saliva proteins.pdf]

SPOT	UniProt ID[u]	SCORE/ MATCHES	SEQ. COV.	PROTEIN	PROTEIN CLASS[p]	LOCALIZATION[u]	MOLECULAR FUNCTION[u]	BIOLOGICAL PROCESS[u]
a1	P02111 (HBB_ORNAN)	94/6	60%	**Hemoglobin subunit beta**	**Plasma proteins**,<br>Predicted intracellular proteins,<br>Transporters,<br>Disease related genes,<br>FDA approved drug targets	**INTRACELLULAR** (cytosol, erythrocytes)	Heme binding<br>Metal ion binding<br>Oxygen binding<br>Oxygen carrier activity	Oxygen transport<br>Transport
a3	P0DM93 (APOA2_LEPWE)	56/4	38%	**Apolipoprotein A-II**	**Plasma proteins**,<br>Predicted secreted proteins,<br>Predicted intracellular proteins,<br>Cancer-related genes,<br>Candidate cardiovascular disease genes,	**EXTRACELLULAR** (secreted)	Lipid binding	Lipid transport<br>Lipoprotein metabolism
a9	Q9GK13 (MUTA_BOVIN)	60/6	18%	**Methylmalonyl-CoA mutase, mitochondrial**	**Plasma proteins**,<br>Predicted intracellular proteins,<br>Disease related genes,<br>Enzymes,<br>FDA approved drug targets	**INTRACELLULAR** (mitochondrion matrix)	GTPase activity<br>Cobalamin binding<br>Metal ion binding<br>Catalytic activity	Isomerase<br>Metabolism
a16	Q9GK13 (MUTA_BOVIN)	88/10	21%	**Methylmalonyl-CoA mutase, mitochondrial**	**Plasma proteins**,<br>Predicted intracellular proteins,<br>Disease related genes,<br>Enzymes,<br>FDA approved drug targets	**INTRACELLULAR** (mitochondrion matrix)	GTPase activity<br>Cobalamin binding<br>Metal ion binding<br>Catalytic activity	Isomerase<br>Metabolism

SPOT	UniProt ID[u]	SCORE/ MATCHES	SEQ. COV.	PROTEIN	PROTEIN CLASS[p]	LOCALIZATION[u]	MOLECULAR FUNCTION[u]	BIOLOGICAL PROCESS[u]
a42	Q9GK13 (MUTA_BOVIN)	102/8	26%	**Methylmalonyl-CoA mutase, mitochondrial**	**Plasma proteins**, Predicted intracellular proteins, Disease related genes, Enzymes, FDA approved drug targets	**INTRACELLULAR** (mitochondrion matrix)	GTPase activity Cobalamin binding Metal ion binding Catalytic activity	Isomerase Metabolism
a11	Q3ZCI9 (TCPQ_BOVIN)	85/9	20%	**T-complex protein 1 subunit theta**	**Plasma proteins**, Predicted intracellular proteins	**INTRACELLULAR** (cytoskeleton, cytosol)	ATP binding Unfolded protein binding	Protein folding
a13	Q3ZCI9 (TCPQ_BOVIN)	71/9	25%	**T-complex protein 1 subunit theta**	**Plasma proteins**, Predicted intracellular proteins	**INTRACELLULAR** (cytoskeleton, cytosol)	ATP binding Unfolded protein binding	Protein folding
a12	Q0V8H6 (MOV10_BOVIN)	82/11	13%	**Putative helicase MOV-10**	**Plasma proteins**, Predicted intracellular proteins, Enzymes,	**INTRACELLULAR** (cytosol)	ATP binding Helicase activity RNA binding	miRNA-mediated gene silencing mRNA cleavage
a14	M3XYN3 (APOA1_MUSPF)	67/4	19%	**Apolipoprotein A-I**	**Plasma proteins**, Predicted secreted proteins, Predicted intracellular proteins, Disease related genes, Cancer-related genes, Candidate cardiovascular disease genes,	**EXTRACELLULAR** (secreted)	Lipid binding	Cholesterol metabolism & transport Lipid metabolism & transport Lipoprotein metabolic process
a17	A4FUD9 (MCM3_BOVIN)	86/10	19%	**Minichromosome maintenance complex component 3**	**Plasma proteins**, Predicted intracellular proteins, Cancer-related genes,	**INTRACELLULAR** (nucleus)	ATP binding DNA binding Helicase activity	Cell cycle DNA replication initiation

SPOT	UniProt ID[u]	SCORE/ MATCHES	SEQ. COV.	PROTEIN	PROTEIN CLASS[p]	LOCALIZATION[u]	MOLECULAR FUNCTION[u]	BIOLOGICAL PROCESS[u]
					Enzymes,			
a19	P11974 (KPYM_RABIT)	90/8	19%	**Pyruvate kinase PKM**	**Plasma proteins**, Predicted intracellular proteins, Cancer-related genes, Enzymes,	**INTRACELLULAR** (cytosol)	Kinase activity ATP binding Magnesium & potassium ion binding	Glycolysis
a20	P08907 (AATM_HORSE)	59/13	27%	**Aspartate aminotransferase, mitochondrial**	**Plasma proteins**, Predicted secreted proteins, Enzymes, Transporters, Cancer-related genes, Candidate cardiovascular disease genes,	**INTRACELLULAR** (mitochondrion matrix)	Transferase activity Pyridoxal phosphate binding	Aspartate metabolic process Biosynthetic process
a22	Q6UR05 (MRP1_CANLF)	99/15	12%	**Multidrug resistance-associated protein 1**	**Plasma proteins**, Predicted membrane proteins, Cancer-related genes, Transporters, FDA approved drug targets,	**INTRACELLULAR** (cell membrane)	ATP binding ATPase activity Transporter activity	Drug/Xenobiotic transmembrane transport Glutathione transmembrane transport
a31	P07318 (CRBB1_BOVI N)	76/7	30%	**Beta-crystallin B1**	**Plasma proteins**, Predicted intracellular proteins, Disease related genes,	**INTRACELLULAR**	Structural constituent of eye lens	Visual perception
a57	E1BPX4 (MCM8_BOVI N)	85/9	16%	**DNA helicase MCM8**	**Plasma proteins**, Predicted intracellular proteins, Enzymes,	**INTRACELLULAR** (nucleus)	ATP binding DNA binding Helicase activity	Cell cycle Initiation of replication DNA repair

SPOT	UniProt ID[u]	SCORE/ MATCHES	SEQ. COV.	PROTEIN	PROTEIN CLASS[p]	LOCALIZATION[u]	MOLECULAR FUNCTION[u]	BIOLOGICAL PROCESS[u]
					Disease related genes, Potential drug targets,			Gamete generation
a59	P02769 (ALBU_BOVIN)	83/16	30%	**Serum albumin**	**Plasma proteins**, Predicted secreted proteins, Predicted intracellular proteins, Cancer-related genes, Candidate cardiovascular disease genes, Disease related genes,	**EXTRACELLULAR** (secreted)	Substance binding & transport	Blood transport Oncotic pressure maintance
a60	P02769 (ALBU_BOVIN)	78/17	28%	**Serum albumin**	**Plasma proteins**, Predicted secreted proteins, Predicted intracellular proteins, Cancer-related genes, Candidate cardiovascular disease genes, Disease related genes,	**EXTRACELLULAR** (secreted)	Substance binding & transport	Blood transport Oncotic pressure maintance
a25	P07435 (OBP_BOVIN)	60/6	44%	**Odorant-binding protein**	**Predicted secreted proteins**,	**EXTRACELLULAR** (secreted)	Small molecule binding	Response to stimulus Sensory perception of smell
a33	P07435 (OBP_BOVIN)	69/10	55%	**Odorant-binding protein**	**Predicted secreted proteins**,	**EXTRACELLULAR** (secreted)	Small molecule binding	Response to stimulus Sensory perception of

SPOT	UniProt ID[u]	SCORE/ MATCHES	SEQ. COV.	PROTEIN	PROTEIN CLASS[p]	LOCALIZATION[u]	MOLECULAR FUNCTION[u]	BIOLOGICAL PROCESS[u]
								smell
a45	P79124 (SPL2A_BOVIN)	65/10	61%	**Short palate, lung and nasal epithelium carcinoma-associated protein 2A**	**Predicted secreted proteins**,	**EXTRACELLULAR** (secreted)	Lipid binding	Unknown
a47	Q1LZC5 (ANR54_BOVI N)	70/7	15%	**Ankyrin repeat domain-containing protein 54**	**Predicted secreted proteins**, Predicted intracellular proteins,	**INTRACELLULAR** (cytosol, nucleus, secreted)	Protein-containing complex binding Protein kinase regulator activity	Nucleo-cytoplasmic transport Regulation of erythrocyte differentiation Intracellular signal transduction
a56	P80025 (PERL_BOVIN)	83/10	14%	**Lactoperoxidase**	**Predicted secreted proteins**, Predicted intracellular proteins, Enzymes,	**EXTRACELLULAR** (milk, saliva) **INTRACELLULAR** (cytosol of salivary gland)	Calcium ion binding Heme binding Thiocyanate peoxidase activity	Antibacterial humoral response Response to oxidative stress Sensory perception
a12	Q2HJ63 (CAHM2_BOVI N)	72/7	28%	**Calcium homeostasis modulator protein 2**	**Predicted membrane proteins**, Transporters,	**INTRACELLULAR** (cell membrane)	Cation channel activity	Ion transmembrane transport
a18	Q01888 (GDC_BOVIN)	84/8	26%	**Graves disease carrier protein**	**Predicted membrane proteins**, Predicted intracellular proteins, Transporters,	**INTRACELLULAR** (mitochondrion inner membrane)	Transmembrane transporter activity	Mitochondrial transport
a26	Q29RT8	60/4	17%	**Phospholipid**	**Predicted membrane**	**INTRACELLULAR**	Lipid phosphatase	Phospholipid

SPOT	UniProt ID[u]	SCORE/ MATCHES	SEQ. COV.	PROTEIN	PROTEIN CLASS[p]	LOCALIZATION[u]	MOLECULAR FUNCTION[u]	BIOLOGICAL PROCESS[u]
	(PLPR2_BOVIN)			**phosphatase-related protein type 2**	**proteins**,<br>Enzymes,	(cell membrane)	activity<br>Phosphatide phosphatase activity	dephosphorylation<br>Phospholipid metabolism<br>Signal transduction
a41	Q95L74 (CY24B_BISBI)	75/8	18%	**Cytochrome b-245 heavy chain**	**Predicted membrane proteins,**<br>Transporters,<br>Disease related genes,<br>Potential drug targets,	**INTRACELLULAR** (plasma membrane in subsets of immune cells)	Metal ion binding<br>Oxidoreductase activity<br>Voltage-gated ion channel activity	Immune response<br>Transmembrane transport (ions and electrons)
a50	Q7YS88 (RAMP3_PIG)	84/7	17%	**Receptor activity-modifying protein 3**	**Predicted membrane proteins**,<br>FDA approved drug targets,	**INTRACELLULAR** (cell membrane)	Protein transporter activity	Receptor<br>Intracellular protein transport
a2	A4IFI1 (CC157_BOVIN)	97/9	19%	**Coiled-coil domain-containing protein 157**	**Predicted intracellular proteins**,	**INTRACELLULAR**	Unknown	Unknown
a8	A4IFI1 (CC157_BOVIN)	133/13	23%	**Coiled-coil domain-containing protein 157**	**Predicted intracellular proteins**,	**INTRACELLULAR**	Unknown	Unknown
a21	A4IFI1 (CC157_BOVIN)	65/8	14%	**Coiled-coil domain-containing protein 157**	**Predicted intracellular proteins**,	**INTRACELLULAR**	Unknown	Unknown
a4	Q29460 (PA1B3_BOVIN)	66/6	23%	**Platelet-activating factor acetylhydrolase IB subunit gamma**	**Predicted intracellular proteins**,<br>Enzymes,	**INTRACELLULAR** (cytoplasmic and membranous expression in several tissues, including subsets	Esterase activity<br>Identical protein binding<br>Platelet-activating factor acetyltransferase	Brain development<br>Lipid catabolic process<br>Spermatogenesis

SPOT	UniProt ID[u]	SCORE/ MATCHES	SEQ. COV.	PROTEIN	PROTEIN CLASS[p]	LOCALIZATION[u]	MOLECULAR FUNCTION[u]	BIOLOGICAL PROCESS[u]
						of immune cells)	activity	
a6	Q2TBL4 (RPC3_BOVIN)	84/9	27%	**DNA-directed RNA polymerase III subunit RPC3**	**Predicted intracellular proteins,** RNA polymerase related proteins,	**INTRACELLULAR** (nucleus)	RNA polymerase activity Single-stranded DNA binding	Transcription Immune response
a7	Q29RJ2 (TBC24_BOVIN)	73/6	21%	**TBC1 domain family member 24**	**Predicted intracellular proteins**, Disease related genes,	**INTRACELLULAR** (cytosol)	GTPase activation	Unknown
a10	P79106 (PAFA2_BOVI N)	74/9	22%	**Platelet-activating factor acetylhydrolase 2, cytoplasmic**	**Predicted intracellular proteins**, Enzymes,	**INTRACELLULAR** (ER membrane)	Esterase activity	Lipid catabolic process
a13	P79106 (PAFA2_BOVI N)	78/8	23%	**Platelet-activating factor acetylhydrolase 2, cytoplasmic**	**Predicted intracellular proteins**, Enzymes,	**INTRACELLULAR** (ER membrane)	Esterase activity	Lipid catabolic process
a15	Q0VC16 (TGO1_BOVIN)	60/13	8%	**Transport and Golgi organization protein 1 homolog**	**Predicted intracellular proteins**,	**INTRACELLULAR** (ER membrane)	Lipoprotein trasporter activity	ER-Golgi transport (lipoprotein, protein transport) Exocytosis
a23	O97557 (TUFT1_PIG)	58/11	47%	**Tuftelin**	**Predicted intracellular proteins**,	**INTRACELLULAR** (cytosol, secreted)	Unknown	Biomineralizatio n Biomineral tissue development
a24	Q32KZ9 (TEKT1_BOVIN)	112/11	35%	**Tektin-1**	**Predicted intracellular proteins**,	**INTRACELLULAR** (cytoskeleton)	Unknown	Cilium assembly Cilium movement involved in cell motility

SPOT	UniProt ID[u]	SCORE/ MATCHES	SEQ. COV.	PROTEIN	PROTEIN CLASS[p]	LOCALIZATION[u]	MOLECULAR FUNCTION[u]	BIOLOGICAL PROCESS[u]
								Flagellated sperm motility
a27	Q17R01 (FXL14_BOVIN)	62/6	33%	**F-box/LRR-repeat protein 14**	**Predicted intracellular proteins**,	**INTRACELLULAR** (cytosol)	Ubiquitin-protein transferase avtivity	Ubiquitin-dependent protein catabolic process
a28	A4FUG8 (RABE2_BOVI N)	78/9	19%	**Rab GTPase-binding effector protein 2 (Rabaptin)**	**Predicted intracellular proteins**,	**INTRACELLULAR** (endosome, cytosol)	Growth factor activity<br>GTPase activator activity	Endocytosis<br>Protein transport
a32	A5D7B1 (RSAD1_BOVI N)	79/9	25%	**Radical S-adenosyl methionine domain-containing protein 1, mitochondrial**	**Predicted intracellular proteins**,	**INTRACELLULAR** (mitochondrion)	Metal ion binding<br>Oxidoreductase activity	Porphyrin-containing compound biosyntesis
a37	Q1JQ97 (BBS4_BOVIN)	70/7	20%	**Bardet-Biedl syndrome 4 protein homolog**	**Predicted intracellular proteins**,<br>Disease related genes,	**INTRACELLULAR** (cytoskeleton, cell membrane, cytosol of lymphoid cells)	Alpha & beta tubulin binding<br>Dynacting binding<br>Microtubule motor activity	Cilium biogenesis/degradation<br>Protein transport
a38	P13214 (ANXA4_BOVI N)	64/14	46%	**Annexin A4**	**Predicted intracellular proteins**,<br>Cancer-related genes,	**INTRACELLULAR** (cell membrane, cytosol, nucleus of epithelial tissues)	Calcium-dependent protein & phospholipid binding<br>Calcium ion binding<br>Carbohydrate, chondroitin sulphate and	Epithelial cell differentiation<br>Notch signaling pathway<br>Regulation of transcription

SPOT	UniProt ID[u]	SCORE/ MATCHES	SEQ. COV.	PROTEIN	PROTEIN CLASS[p]	LOCALIZATION[u]	MOLECULAR FUNCTION[u]	BIOLOGICAL PROCESS[u]
							heparin binding	
a40	Q2KJ25 (PSD12_BOVIN)	118/12	35%	**26S proteasome non-ATPase regulatory subunit 12**	**Predicted intracellular proteins**,	**INTRACELLULAR** (cytosol, nucleus)	Unknown	Proteasome-mediated ubiquitin-dependent protein catabolic process
a51	Q2HJG4 (TDRD3_BOVIN)	89/10	17%	**Tudor domain-containing protein 3**	**Predicted intracellular proteins**,	**INTRACELLULAR** (nucleus, cytosol)	Chromatin binding Methylated histone binding Transcription coactivator activity	Chromatin organization (chromatin regulator)

[U] – data obtained from https://www.uniprot.org/

[P] – data obtained from https://www.proteinatlas.org/

Matches - the number of peptides in the MS spectrum that match the sequences from the database.

SEQ COV = sequence coverage - what percentage of the protein found in the study is covered by peptides from the MS spectrum.
