# Supplementary material for: Comparative Analysis of Saliva and Plasma Proteins Patterns in Pregnant Cows—Preliminary Studies
Source: Animals (Basel). 2022 Oct 20;12(20):2850. doi: 10.3390/ani12202850 (PMC9597767; doi:10.3390/ani12202850)
Supplement: Supplementary file 1 [file animals-12-02850-s001.zip › Table S2 Identification of plasma proteins.pdf]

SPOT	UniProt ID[u]	SCORE/ MATCHES	SEQ. COV.	PROTEIN	PROTEIN CLASS[p]	LOCALIZATION[u]	MOLECULAR FUNCTION[u]	BIOLOGICAL PROCESS[u]
b24	Q27954 (COPA_BOVIN)	120/17	12%	**Coatomer subunit alpha**	**Plasma proteins**, Predicted intracellular proteins, Disease related genes,	**INTRACELLULAR** (Golgi apparatus membrane) **EXTRACELLULAR** (secreted)	Hormone activity Structural molecule activity	Intracellular protein transport (ER - Golgi vesicle-mediated transport)
b34	Q58DG6 (FXL20_BOVIN)	96/9	20%	**F-box/LRR-repeat protein 20**	**Plasma proteins**, Predicted intracellular proteins,	**INTRACELLULAR** (cytosol)	Unknown	behavioral fear response ubiquitin conjugation pathway
b35	Q9GK13 (MUTA_BOVIN)	104/11	26%	**Methylmalonyl-CoA mutase, mitochondrial**	**Plasma proteins**, Predicted intracellular proteins, Disease related genes, Enzymes, FDA approved drug targets,	**INTRACELLULAR** (mitochondrion matrix)	GTPase activity Cobalamin binding Metal ion binding Catalytic activity	Isomerase Metabolism
b45	Q29RR5 (TFP11_BOVIN)	72/10	13%	**Tuftelin-interacting protein 11**	**Plasma proteins**, Predicted intracellular proteins,	**INTRACELLULAR** (nucleus, cytosol)	Nucleic acid binding	Biomineralization Spliceosomal complex disassembly
b49	P0DM93 (APOA2_LEPWE)	60/3	37%	**Apolipoprotein A-II**	**Plasma proteins**, Predicted secreted proteins, Predicted intracellular proteins, Cancer-related genes, Candidate	**EXTRACELLULAR** (secreted)	Lipid binding	Lipid transport Lipoprotein metabolism

SPOT	UniProt ID[u]	SCORE/ MATCHES	SEQ. COV.	PROTEIN	PROTEIN CLASS[p]	LOCALIZATION[u]	MOLECULAR FUNCTION[u]	BIOLOGICAL PROCESS[u]
					cardiovascular disease genes,			
b50	P68226 (HBB_LAMGL)	56/5	49%	**Hemoglobin subunit beta**	**Plasma proteins**, Predicted intracellular proteins, Transporters, Disease related genes, FDA approved drug targets,	**INTRACELLULAR** (cytosol, erythrocytes)	Heme binding Metal ion binding Oxygen binding Oxygen carrier activity	Oxygen transport Transport
b54	Q5E9B1 (LDHB_BOVIN)	74/7	21%	**L-lactate dehydrogenase B chain**	**Plasma proteins**, Predicted intracellular proteins, Enzymes, Disease related genes, Potential drug targets,	**INTRACELLULAR** (cytosol)	Oxidoredustase activity	Carbohydrate metabolic process
b54	Q3ZBW4 (PCNA_BOVIN)	71/6	20%	**Proliferating cell nuclear antigen**	**Plasma proteins**, Predicted intracellular proteins, Disease related genes, Cancer-related genes,	**INTRACELLULAR** (nucleus)	Chromatin binding DNA binding	DNA replication DNA repair
b55	Q0V8H6 (MOV10_BOVIN)	68/10	12%	**Putative helicase MOV-10**	**Plasma proteins**, Predicted intracellular proteins, Enzymes,	**INTRACELLULAR** (cytosol)	ATP binding Helicase activity RNA binding	miRNA-mediated gene silencing mRNA cleavage
b57	P81425	97/11	13%	**Dipeptidyl peptidase 4**	**Plasma proteins**,	**INTRACELLULAR**	Catalytic	Cell adhesion

SPOT	UniProt ID[u]	SCORE/ MATCHES	SEQ. COV.	PROTEIN	PROTEIN CLASS[p]	LOCALIZATION[u]	MOLECULAR FUNCTION[u]	BIOLOGICAL PROCESS[u]
	(DPP4_BOVIN)				Predicted intracellular proteins,<br>Predicted membrane proteins,<br>Enzymes,<br>FDA approved drug targets,<br>CD markers,	(cell membrane)<br>**EXTRACELLULAR** (secreted)	activity<br>Signalling receptor binding	Endothelial cell migration<br>T-cell costimulation
b58	B2D1U1 (CBG_URSAR)	73/6	21%	**Corticosteroid-binding globulin**	**Plasma proteins**,<br>Predicted secreted proteins,<br>Disease related genes,	**EXTRACELLULAR** (secreted)	Steroid binding	Transport
b60	P82533 (CRYAA_ERIEU)	61/3	14%	**Alpha-crystallin A chain**	**Plasma proteins**,<br>Predicted intracellular proteins,<br>Disease related genes,	**INTRACELLULAR** (nucleus, cytosol)	Metal ion binding<br>Eye lens structure	Eye lens structure
b61	O46375 (TTHY_BOVIN)	80/4	46%	**Transthyretin**	**Plasma proteins**,<br>Predicted secreted proteins,<br>Disease related genes,<br>Cancer-related genes,	**EXTRACELLULAR** (Secreted)	Hormone activity<br>Thyroid hormone binding	Thyroid hormone transport
b62	P15497 (APOA1_BOVIN)	245/22	68%	**Apolipoprotein A-I**	**Plasma proteins**,<br>Predicted secreted proteins,<br>Predicted intracellular proteins,<br>Disease related genes,<br>Cancer-related genes,	**EXTRACELLULAR** (secreted)	Lipid binding	Cholesterol metabolism & transport<br>Lipid metabolism & transport<br>Lipoprotein

SPOT	UniProt ID[u]	SCORE/ MATCHES	SEQ. COV.	PROTEIN	PROTEIN CLASS[p]	LOCALIZATION[u]	MOLECULAR FUNCTION[u]	BIOLOGICAL PROCESS[u]
					Candidate cardiovascular disease genes,			metabolic process
b63	P15497 (APOA1_BOVIN)	252/22	60%	**Apolipoprotein A-I**	**Plasma proteins**, Predicted secreted proteins, Predicted intracellular proteins, Disease related genes, Cancer-related genes, Candidate cardiovascular disease genes,	**EXTRACELLULAR** (secreted)	Lipid binding	Cholesterol metabolism & transport Lipid metabolism & transport Lipoprotein metabolic process
b64	P15497 (APOA1_BOVIN)	147/16	52%	**Apolipoprotein A-I**	**Plasma proteins**, Predicted secreted proteins, Predicted intracellular proteins, Disease related genes, Cancer-related genes, Candidate cardiovascular disease genes,	**EXTRACELLULAR** (secreted)	Lipid binding	Cholesterol metabolism & transport Lipid metabolism & transport Lipoprotein metabolic process
b30	P10522 (MYP0_BOVIN)	86/8	21%	**Myelin protein P0**	**Predicted membrane proteins** , Predicted intracellular proteins, Disease related genes,	**INTRACELLULAR** (cell membrane, myelin sheath)	Unknown	Cell agregation Cell-to-celladhesion Myelination
b33	Q2HJB9 (TMM98_BOVIN)	90/6	30%	**Transmembrane protein 98**	**Predicted membrane proteins**, Disease related genes,	**INTRACELLULAR** (cell membrane)	Unknown	Unknown

SPOT	UniProt ID[u]	SCORE/ MATCHES	SEQ. COV.	PROTEIN	PROTEIN CLASS[p]	LOCALIZATION[u]	MOLECULAR FUNCTION[u]	BIOLOGICAL PROCESS[u]
b25	A1A4L4 (MINY4_BOVIN)	92/11	15%	**Probable ubiquitin carboxyl-terminal hydrolase MINDY-4**	**Predicted intracellular proteins**, Enzymes,	**INTRACELLULAR** (cytosol, cell membrane)	Thiol-dependent ubiquitinyl hydrolase activity	Ubiquitin conjugation pathway
b26	Q5E9F9 (PRS7_BOVIN)	60/13	33%	**26S proteasome regulatory subunit 7**	**Predicted intracellular proteins**,	**INTRACELLULAR** (nucleus, cytosol)	Proteasome-activating ATPase activity ATP binding	Ubiquitin-dependent protein catabolic pathway
b27	Q0VBY1 (ODF2L_BOVIN)	63/9	18%	**Outer dense fiber protein 2-like**	**Predicted intracellular proteins**,	**INTRACELLULAR** (cytosol, mitochondrion)	unknown	Cell projection organization Negative regulation of cilium assembly
b53	Q0VBY1 (ODF2L_BOVIN)	76/7	17%	**Outer dense fiber protein 2-like**	**Predicted intracellular proteins**,	**INTRACELLULAR** (cytosol, mitochondrion)	unknown	Cell projection organization Negative regulation of cilium assembly
b29	Q2MJS5 (MT3_BOSMU)	59/4	72%	**Metallothionein-3**	**Predicted intracellular proteins,**	**INTRACELLULAR** (nucleus, synaptic vesicles)	Heavy metals ion binding Drug binding Protein kinase activity	Astrocyte development Heavy ions homeostasis
b43	Q5E977 (F118B_BOVIN)	75/8	14%	**Protein FAM118B**	**Predicted intracellular proteins,**	**INTRACELLULAR** (nucleus)	Unknown	Cajal body organization

SPOT	UniProt ID[u]	SCORE/ MATCHES	SEQ. COV.	PROTEIN	PROTEIN CLASS[p]	LOCALIZATION[u]	MOLECULAR FUNCTION[u]	BIOLOGICAL PROCESS[u]
b44	A5PK00 (VPS36_BOVIN)	104/10	32%	**Vacuolar protein-sorting-associated protein 36**	**Predicted intracellular proteins,**	**INTRACELLULLAR** (nucleus, endosome in most tissues)	Lipid binding Ubiquitin binding	Protein transport Transcription Regulation of transcription
b46	P46446 (ACY2_BOVIN)	72/7	20%	**Aspartoacylase**	**Predicted intracellular proteins,** Disease related genes, Enzymes, Potential drug targets,	**INTRACELLULAR** (nucleus, cytosol)	Metal ion binding Hydrolase activity	Metabolic process
b47	Q5E9B4 (EI2BB_BOVIN)	81/8	23%	**Translation initiation factor eIF-2B subunit beta**	**Predicted intracellular proteins,** Disease related genes,	**INTRACELLULAR** (cytosol)	ATP, GTP binding Translation initiation factor activity	Central nervous system development Myelination Ovarian development T-cell receptor signalling pathway Translation initiation (protein biosyntesis)
b48	Q3ZC08 (ANXA9_BOVI N)	95/8	31%	**Annexin A9**	**Predicted intracellular proteins,**	**INTRACELLULAR** (cell membrane, cytosol)	Calcium ion binding Calcium-dependent phospholipid binding	Low affinity receptor for acetylcholine
b52	Q2T9L9	60/4	14%	**General transcription**	**Predicted intracellular**	**INTRACELLULAR**	ATP binding	Transcription

SPOT	UniProt ID[u]	SCORE/ MATCHES	SEQ. COV.	PROTEIN	PROTEIN CLASS[p]	LOCALIZATION[u]	MOLECULAR FUNCTION[u]	BIOLOGICAL PROCESS[u]
	(T2FB_BOVIN)			**factor IIF subunit 2**	**proteins,** Enzymes,	(nucleus)	DNA binding Helicase activity	regulation Trancription initiation
b55	Q32L18 (BTBDG_BOVIN)	94/9	23%	**BTB/POZ domain-containing protein 16**	**Predicted intracellular proteins,**	**INTRACELLULAR** (nucleus)	Unknown	Unknown
b60	A4FV72 (PPIE_BOVIN)	73/6	13%	**Peptidyl-prolyl cis-trans isomerase E**	**Predicted intracellular proteins,** Enzymes,	**INTRACELLULAR** (nucleus)	Cyclosporin A binding mRNA binding Catalytic activity	mRNA splicing Protein folding Isomerisation

[U] – data obtained from https://www.uniprot.org/

[P] – data obtained from https://www.proteinatlas.org/

Matches - the number of peptides in the MS spectrum that match the sequences from the database.

SEQ COV = sequence coverage - what percentage of the protein found in the study is covered by peptides from the MS spectrum.
