# Supplementary material for: Comparative Analysis of Saliva and Plasma Proteins Patterns in Pregnant Cows—Preliminary Studies
Source: Animals (Basel). 2022 Oct 20;12(20):2850. doi: 10.3390/ani12202850 (PMC9597767; doi:10.3390/ani12202850)
Supplement: Supplementary file 1 [file animals-12-02850-s001.zip › Table S3 Identification of proteins from albumin-free fraction.pdf]

SPOT	UniProt ID[u]	SCORE/ MATCHES	SEQ. COV.	PROTEIN	PROTEIN CLASS[p]	LOCALIZATION[u]	MOLECULAR FUNCTION[u]	BIOLOGICAL PROCESS[u]
c3	Q3ZCI9 (TCPQ_BOVIN)	119/11	26%	**T-complex protein 1 subunit theta**	**Plasma proteins**,<br>Predicted intracellular proteins,	**INTRACELLULAR** (cytoskeleton, cytosol)	ATP binding<br>Unfolded protein binding	Protein folding
c4	Q2KJF1 (A1BG_BOVIN)	93/13	48%	**Alpha-1B-glycoprotein**	**Plasma proteins**,<br>Predicted intracellular proteins,<br>Predicted secreted proteins,	**EXTRACELLULAR** (secreted - liver)	Unknown	Platelet degranulation<br>Neutrophil degranulation
c12	Q95215 (BGH3_RABIT)	99/10	21%	**Transforming growth factor-beta-induced protein ig-h3**	**Plasma proteins**,<br>Predicted intracellular proteins,<br>Predicted secreted proteins<br>Disease related genes	**EXTRACELLULAR** (secreted)	Collagen binding<br>Extracellular matrix binding<br>Integrin binding	Cell adhesion
c19	Q9GK13 (MUTA_BOVIN)	58/14	28%	**Methylmalonyl-CoA mutase, mitochondrial**	**Plasma proteins**,<br>Predicted intracellular proteins,<br>Disease related genes,<br>Enzymes,<br>FDA approved drug targets	**INTRACELLULAR** (mitochondrion matrix)	GTPase activity<br>Cobalamin binding<br>Metal ion binding<br>Catalytic activity	Isomerase<br>Metabolism
c23	P27124 (FKBP4_RABIT)	97/9	31%	**Peptidyl-prolyl cis-trans isomerase FKBP4**	**Plasma proteins**,<br>Predicted intracellular proteins,	**INTRACELLULAR** (nucleus, cytosol, cytoskeleton and mitochondrion)	ATP & GTP binding<br>Glucocorticoid receptor binding<br>Heat shock protein binding<br>Isomerase activity	Androgen receptor signalling pathway<br>Protein folding<br>Embryo implantation & male sex differentiation

SPOT	UniProt ID[u]	SCORE/ MATCHES	SEQ. COV.	PROTEIN	PROTEIN CLASS[p]	LOCALIZATION[u]	MOLECULAR FUNCTION[u]	BIOLOGICAL PROCESS[u]
								Steroid hormone receptor complex assembly
c25	Q9TTC6 (PPIA_RABIT)	70/5	28%	**Peptidyl-prolyl cis-trans isomerase A**	**Plasma proteins**, Predicted intracellular proteins, Enzymes,	**EXTRACELLULAR** (secreted - cytosol, nucleus)	Cyclosporine A binding Isomerase activity	Protein folding Protein isomerisation
c27	P08831 (IL1A_BOVIN)	66/7	27%	**Interleukin-1 alpha**	**Plasma proteins**, Predicted intracellular proteins, Transporters, Cancer-related genes,	**EXTRACELLULAR** (secreted – cytosol)	Cooper ion binding Cytokine activity	Cytokine-mediated signaling pathway Inflamatory response Pyrogen
c27	Q3T0P6 (PGK1_BOVIN)	64/7	25%	**Phosphoglycerate kinase 1**	**Plasma proteins**, Predicted intracellular proteins, Disease related genes, Enzymes, Potential drug targets	**INTRACELLULAR** (cytosol)	ATP binding Catalytic activity	Glycolytic process Epithelial cell differentiation Response to hypoxia
c21	Q0VCP3 (OLFL3_BOVIN)	62/6	17%	**Olfactomedin-like protein 3**	**Predicted secreted proteins** , Predicted intracellular proteins,	**EXTRACELLULAR** (secreted)	Unknown	Multicellular organizm development (development al protein)
c7	Q3SZE9 (TBCC_BOVIN)	76/7	29%	**Tubulin-specific chaperone C**	**Predicted intracellular proteins,**	**INTRACELLULAR** (cytosol)	GTPase activity Tubulin binding	Cell morphogenesi s

SPOT	UniProt ID[u]	SCORE/ MATCHES	SEQ. COV.	PROTEIN	PROTEIN CLASS[p]	LOCALIZATION[u]	MOLECULAR FUNCTION[u]	BIOLOGICAL PROCESS[u]
								Tubulin complex assembly<br>Protein folding
c18	A5PK00 (VPS36_BOVIN)	116/10	38%	**Vacuolar protein-sorting-associated protein 36**	**Predicted intracellular proteins**,	**INTRACELLULLAR** (nucleus, endosome)	Lipid binding<br>Ubiquitin binding	Protein transport (to vacuole involved in ubiquitin-dependent protein catabolic process)<br>Transcription<br>Regulation of transcription
c20	P05128 (KPCG_BOVIN)	106/9	22%	**Protein kinase C gamma type**	**Predicted intracellular proteins**,<br>Disease related genes,<br>Enzymes,<br>Potential drug targets,<br>RAS pathway related proteins	**INTRACELLULAR** (cell membrane, cytosol)	ATP binding<br>Protein kinase activity<br>Zinc ion binding	Catalytic activity<br>Intracellular signal transduction<br>Biological rhythms
c22	A4IFK7 (RIPL2_BOVIN)	67/6	34%	**RILP-like protein 2 (Rab Interacting Lysosomal Protein – like 2)**	**Predicted intracellular proteins**,	**INTRACELLULAR** (cytoskeleton, cytosol, cilium)	Dynein light intermediate chain binding<br>Small GTPase binding<br>Protein dimerization activity	Cilium assembly<br>Epithelial cell morphogenesis<br>Protein transport (from ciliary

SPOT	UniProt ID[u]	SCORE/ MATCHES	SEQ. COV.	PROTEIN	PROTEIN CLASS[p]	LOCALIZATION[u]	MOLECULAR FUNCTION[u]	BIOLOGICAL PROCESS[u]
								membrane to plasma membrane)
c24	A6QL79 (NATD1_BOVIN)	64/4	53%	**Protein NATD1 (N-acetyltransferase domain-containing protein 1)**	**Predicted intracellular proteins**,	**INTRACELLULAR**	Unknown	Unknown
c26	E1BLZ4 (ZFY26_BOVIN)	105/15	9%	**Zinc finger FYVE domain-containing protein 26**	**Predicted intracellular proteins**, Disease related genes,	**INTRACELLULAR** (cytoskeleton, cytosol)	Metal ion binding PIP3 binding	Cell cycle Cell division Regulation of cytokinesis DNA repair
c31	O97725 (NDUAC_BOVIN)	81/5	46%	**NADH dehydrogenase [ubiquinone] 1 alpha subcomplex subunit 12**	**Predicted intracellular proteins**, Disease related genes,	**INTRACELLULAR** (mitochondrion inner membrane)	Electron transfer activity	Electron transport Respiratory chain Mitochondrial ATP synthesis
c32	E1BFE9 (BI2L2_BOVIN)	62/7	17%	**Brain-specific angiogenesis inhibitor 1-associated protein 2-like protein 2**	**Predicted intracellular proteins**,	**INTRACELLULAR** (cell membrane)	Phospholipid binding	Actin crosslink formation Insulin receptor signalling pathway Actin filament organisation

[U] – data obtained from https://www.uniprot.org/

[P] – data obtained from https://www.proteinatlas.org/

Matches - the number of peptides in the MS spectrum that match the sequences from the database.

SEQ COV = sequence coverage - what percentage of the protein found in the study is covered by peptides from the MS spectrum.
